# Monogenic polyarteritis nodosa caused by ADA2 Deficiency: the GOSH experience

**DOI:** 10.1186/1546-0096-13-S1-P8

**Published:** 2015-09-28

**Authors:** S Nanthapisal, C Murphy, E Omoyinmi, A Standing, Y Hong, SM Gomes, N Klein, D Eleftheriou, PA Brogan

**Affiliations:** 1 UCL Institute of Child Health, 30 Guilford Street, UK

## Introduction

Recessive mutations in Cat Eye syndrome Critical Region 1 (*CECR1*), the gene encoding adenosine deaminase 2 (ADA2) have been recently reported to cause polyarteritis nodosa (PAN) with highly varied clinical expression.

## Objectives

The aim of this study was to identify the relevance of this mutation in children with PAN at Great Ormond Street Hospital (GOSH).

## Patients and methods

We used next generation (NGS) and Sanger sequencing to study select paediatric cases of PAN. Inclusion criteria were: 1. Onset of PAN <age-10-years; 2. Suspected familial PAN; 3. Sporadic PAN particularly with neurological involvement; and 4. Clinical features resembling the recent description of deficiency of ADA2 (DADA2). Whole exome sequencing was performed using a commercially available kit (Illumina) and a NextSeq500 sequencer. We used the Infinium HumanCytoSNP-12 v2.1 DNA Analysis BeadChip Kit for homozygosity mapping. Sanger sequencing was performed using the Applied Biosystems 3730 DNA Analyzer. ADA2 enzymatic assay was performed using Diazyme ADA Test Kit.

## Results

Fourteen patients with PAN and one patient with unclassified vasculitis were included. Mutations in *CECR1* were identified in 8 patients: 4 homozygous mutations and 4 compound heterozygous mutations. Of the 8 patients with *CECR1* mutation, all had the evidence of systemic inflammation; 8/8 had cutaneous vasculitis; 3/8 had arterial ischaemic stroke; 3/8 had vasculitic polyneuropathy; 1/8 had long-tract signs, cause undetermined; and 3/8 had B cell immunodeficiency. Four/8 ultimately required treatment with anti-TNF-alpha having failed other treatments. We also screened 25 subjects from 7 families related to the index cases with DADA2. Homozygous (n=4) or compound heterozygous mutations (n=1) were identified in 5/25 of these asymptomatic subjects. ADA2 enzyme activity in both affected and asymptomatic subjects with *CECR1* mutations was significantly lower compared to healthy paediatric controls (*p* value = 0.0022) and sporadic PAN patients without *CECR1* mutation (*p* value = 0.0189).

## Conclusions

We identified DADA2 as the cause of vasculitis in 8/15 (53%) of cases with childhood PAN; and 5 currently asymptomatic cases in their relatives. The clinical severity of DADA2 is heterogeneous ranging from asymptomatic to full-blown systemic PAN. It is currently unknown how DADA2 causes vasculitis. Possibilities include alteration of macrophage biology; endothelial activation; effect of chronic high extracellular adenosine; and/or other as yet poorly defined immunodysregulation (with or without immunodeficiency). Irrespective of the mechanism, TNF blockade seems to be effective therapeutically. Homozygous symptomatic patients may first present in middle age hence asymptomatic DADA2 requires close monitoring.

